# The impact of obesity to antioxidant defense parameters in adolescents with increased cardiovascular risk

**DOI:** 10.2478/jomb-2019-0051

**Published:** 2020-09-02

**Authors:** Emina Čolak, Dragana Pap, Ljubinka Nikolić, Sanja Vicković

**Affiliations:** 1 University of Belgrade, Clinical Center of Serbia and School of Pharmacy, Institute of Medical Biochemistry; 2 Institute for the Health Protection of Students, Department of Laboratory Diagnostics, Novi Sad; 3 Clinical Center of Serbia, Department for Hematology and Transfusion Laboratory, Clinic for Gynecology and Obstetrics; 4 University of Novi Sad, Clinical Center of Vojvodina and School of Medicine, Department of Anesthesiology and Intensive Care

**Keywords:** obesity, oxidative stress, antioxidant defense, cardiovascular risk factors, inflammation, gojaznost, oksidativni stres, antioksidantna zaštita, kardiovaskularni faktori rizika, inflamacija

## Abstract

**Background:**

The goal of this study was to assess the oxidative stress status through the values of antioxidant defense parameters: superoxide dismutase (SOD), glutathione peroxidase (GPx), glutathione reductase (GR) and total antioxidant status (TAS), as well as cardiovascular risk factors (total cholesterol, LDL-cholesterol, VLDL-cholesterol, non-HDL-cholesterol and triglycerides), anthropometric parameters (Body mass index-BMI, waist circumference-WC, hipp circumferemce-HC, waist-to-hipp ratio-WHR and inflammatory markers (high sensitive C-reactive protein) in a group of obese adolescents.

**Methods:**

A total of 238 students of both sexes, age of 22.32 ± 1.85 yr. were included in the study. According to the values of BMI lower and higher than 25 kg/m2 and WC higher and lower than 94 cm (males)/80 cm (females) the tested group of students was divided into 2 subgroups: Group 1 (increased risk for CVD) and Group 2 (lower risk for CVD).

**Results:**

Significantly reduced SOD and GPx with increased GR, TAS, inflammatory and lipoprotein parameters were obtained in Group 1 compared to Group 2. Significant positive association of hsCRP (OR:1.41; 95%CI 1.08-1.83; P=0.007), TAS (OR:827.2; 95%CI 19.27-35498; P=0.007) and GR (OR:1.13; 95%CI 1.05-1.21; P=0.002) and negative association of GPx (OR:0.97; 95%CI 0.94-1.003; P=0.043) and HDL-cholesterol (OR:0.41; 95%CI 0.176-0.963; P=0.0014) with cardiovascular risk factors were found in obese students. According to the ROC analysis GR>44.8 U/L, TAS>1.35 mmol/L, hsCRP>1.71 mg/L and HDL-cholesterol <1.13 mmol/L have sufficient predictive ability for cardiovascular disease in obese students.

**Conclusions:**

Significant association of antioxidant defense parameters with anthropometric, lipid and inflammatory markers in obese students with increased cardiovascular risk suggest that screening of these parameters is necessary and highly recommended.

## Abstract (sr)

**Uvod:**

Cilj ovog rada je bio da se proceni oksidativni status u grupi gojaznih adolescenata preko vrednosti antioksidantih parametara: superoksid dizmutaze (SOD), glutation peroksidaze (GPx), glutation reduktaze (GR) i totalnog antioksidantnog statusa (TAS), kardiovaskulanih faktora rizika (ukupni holesterol, LDL-holesterol, VLDLholesterol, non-HDL-holesterol, i trigliceridi), antropometrijskih parametara (Body mass index-BMI, obim struka, obim kuka i odnos struk/kuk) i inflamatornog markera visoko osetljivog C-reaktivnog proteina (hsCRP).

**Metode:**

U studiju je uključeno 238 studenata, oba pola, starosti od 22,32±1,85 godina. U odnosu na vrednosti BMI većih ili manjih od 25 kg/m2, i obima struka veće ili manje od 94 cm (za muškarce) tj. 80 cm (za žene), celokupna grupa studenata je podeljena na dve podgrupe: Grupa 1 (sa povećanim kardiovaskularnim rizikom) i Grupa 2 (sa sniženim kardiovaskularnim rizikom).

**Rezultati:**

Značajno smanjene vrednosti SOD-a, GPx-a, a povećane vrednosti GR, TAS-a, inflamatornih i lipidnih parametara su dobijene u Grupi 1 u odnosu na Grupu 2 ispitanika. Dobijena je značajna pozitivna asocijacija hsCRPa (OR:1,41; 95%CI 1,08-1,83; P=0,007), TAS-a (OR:827,2; 95%CI 19,27-35498; P=0.007) GR (OR:1,13; 95%CI 1,05-1,21; P=0,002) i negativna asocijacija GPx-a (OR:0,97; 95%CI 0,94-1,003; P=0,043) i HDL-holesterola (OR:0,41; 95%CI 0,176-0,963; P=0,0014) sa kardiovaskularnim faktorima rizika u grupi gojaznih studenata. ROC analiza je pokazala da su GR>44,8 U/L, TAS>1,35 mmol/L, hsCRP>1,71 mg/L i HDL-holesterol < 1,13 mmol/L dovoljno snažni prediktori kardiovaskularnih bolesti kod gojaznih studenata.

**Zaključak:**

Značajna asocijacija antioksidantnih parametara sa antropometrijskim, lipidnim i inflamatornim markerima u gojaznih studenata ukazuje na to da je skrining ovih parametara neophodan i preporučljiv.

## Introduction

Obesity is defined as a serious nutrition problem, resulting from excessive accumulation of fat. It is documented that abdominal fat is recognized as the major risk factor for obesity related diseases such as: hypertension, dyslipidemia, diabetes mellitus type 2, coronary heart disease, non-alcoholic fatty liver disease, stroke etc. Fat accumulation is associated with increased pro-oxidant and pro-inflammatory states. Recent published studies suggest that oxidative stress could be the link between obesity and related complications [1] [2].

Nowadays, obesity becomes the main cause of diabetes and related diseases. In obese patients occur metabolic changes such as dyslipidemia, insulin resistance, diabetes, fatty-inflammatory degeneration of the liver (non-alcoholic fatty liver disease), kidney and heart failure, cardiomyopathy etc. [3] [4].

Impairment of glucose concentration and insulin function are the cause of the metabolic abnormalities found in obesity. Most recent studies confirm that the BMI values continue to increase all over the world [5]. The reason for this is economic growth and changes in nutrition patterns in combination with available, cheap and unbalanced food, rich in carbohydrates and fat. Excess food intake is usually followed by a sedentary lifestyle, lack of physical activity and increased consumption of high caloric food, nonalcoholic and alcoholic drinks [6].

There are evidences that obesity increasingly affects school children who at young age develop arterial hypertension, cardiovascular disease and diabetes mellitus. Overweight children develop abnormal vascular wall [7]. A recent paper of Niemann et al. concluded that obesity induces premature cardiac aging in young patients [8]. Increased oxidative stress in adipocites is important pathogenic mechanism of metabolic syndrome [9].

It is thought that oxidative stress could be a consequence, but also a trigger of obesity. Increase intake of fats, carbohydrates and saturated fatty acids, especially trans-fatty acids lead to increase oxidative stress. The multiple biochemical mechanisms responsible for increased oxidative stress include several processes such as: the superoxide generation via the activity of NADPH oxidase, oxidative phosphorilation, glyceraldehyde auto-oxidation, protein kinase C (PKC) activation, polyol and hexosamine pathways [10]. On the other hand, oxidative stress plays a causative role in development of obesity by stimulating the deposition of adipose tissue including preadipocite proliferation, adipocite differentiation and growth [11].

Adipose tissue, as an endocrine and energy storage organ, secretes hormones and cytokines (adipokines) which manifest endocrine, paracrine and autocrine action. Adipokines could induce the production of reactive oxygen species. It was documented that oxidative stress is strongly related to inflammatory processes in obesity. Adipose tissue secretes pro-inflammatory citokines such as: tumor-necrosis factor α (TNF-α), interleukin 1β (IL-1β) and IL-6 [12].

The goal of this study was to analyze the oxidative stress status of obese students with and without increased risk for cardiovascular events (CVD) through the values of antioxidant parameters: superoxide dismutase (SOD), glutathione peroxidase (GPx), glutathione reductase (GR) and total antioxidant status (TAS). In addition, the aim was to determine the correlation of tested antioxidant parameters with the cardiovascular risk parameters: total cholesterol, LDL-cholesterol, VLDL-cholesterol, nonHDLcholesterol, triglycerides, high sensitive CRP (hsCRP) and anthropometric parameters as well as: body mass index (BMI), waist circumference (WC), hipp circumference (HC) and waist-to hip ratio (WHR) and to determine the minimum concentration of tested parameters (the cutoff points) that could be associated with cardiovascular risk.

## Materials and Methods

The cross-sectional study, conducted at the Institute for the Health Protection of Students, University of Novi Sad, included a total of 238 students, of both sexes (126 males and 112 females), aged of 18 to 29 yr. (22.32 ± 1.85). They were recruited from a regular medical checkup, from April 2012 to April 2014. Out of total number of tested students, 164 were obese or overweight (BMI>25 kg/m^2^ and WC>94 cm for men, or WC>80 cm for women). They comprised the group with increased risk for CVD (Group 1). The remaining 74 students, having BMI<25 kg/m^2^ and WC<94 cm/< 80 cm for females comprised the control group (Group 2). The blood samples for analysis were taken after 12-14 hours of overnight fast and following parameters were analysed: glucose, lipid levels, superoxide dismutase (SOD), Se-dependent glutathione peroxidase (GPx), glutathione reductase (GR), total antioxidant status (TAS) and hsCRP as well. All tested antioxidant parameters were determined by commercial tests Randox Ltd. UK, based on spectrophotometric methods.

SOD was determined by a method developed by Goldstein [13] in blood hemolysate, obtained by washing of erythrocytes with 154 mmol/L of NaCl. Finally, the aliquots of washed erythrocytes were lysed with cold deionized water and put in a cool place (+ 4 °C) for 15 minutes in order to complete hemolysis. Glutathione peroxidase (GPx) was determined by the method of Paglia and Valentine [14]. According to Miller et al. [15], method for total antioxidant status (TAS) determination included the addition of ABTS ® (2,2'-Azino-di-[3-ethylbenzthiazoline sulphonate)) which helps the visualization of stable blue-green colour, while glutathione reductase (GR) was determined by the method of Goldberg [16]. TAS and GR were determined in plasma, obtained by centrifugation of the Li-heparinized blood during 10 minutes on 3000 rpm. CRP was determined by using an immunochemical high sensitive (hsCRP) method on Olympus AU 400 analyzer.

The lipid parameters (total cholesterol, HDLcholesterol and triglycerides) were assayed by standard laboratory methods on Olympus AU 400 analyzer. HDL-cholesterol was assayed by a direct quantitative method for »in vitro« determination (on Olym pus AU400) using colorimetric, end-point immunoinhibition reaction. VLDL-cholesterol was calculated mathematically using the formula: Triglycerides/2.2. LDLcholesterol was calculated by Friedewald formula. Non-HDL-cholesterol values were obtained mathematically by subtracting HDL-cholesterol from total cholesterol values.

All subjects gave their informed consent to participate in this study which was approved by the local Ethics Committee. For statistical evaluation, the Med Calc statistical package v.9.4.2.0 was used with fol-lowed statistical methods: Student's t-test, Mann-Whitney U test, Chi-Square, and Fisher's exact test. For normally distributed data, results were presented as mean ± standard deviation (SD), while median and interquartile range was used to present the nonnormally distributed data. Spearman's rank and Pearson's correlation test were used to define correlations of the individual parameters between and within the tested groups. All statistical tests were two-tailed and P values 0.05 were considered statistically significant. Logistic regression analysis was used to determine the association of anthropometric with antioxidant and lipoprotein parameters. The diagnostic sensitivity and specificity of the tests and cutoff values associated with cardiovascular risk were calculated by Receiver operating characteristic (ROC) curve. The goal was to determine the cutoff values where area under the curve (AUC) would be higher than 0.5, while sensitivity and/or specificity would be at least 70%.

## Results

Significantly lower SOD (p=0.003) and GPx (p=0.03), higher GR (p=0.0001), TAS (p<0.000) values and lipid parameters: total cholesterol LDLcholesterol, non-HDL-cholesterol, VLDL-cholesterol, and triglycerides (p<0.000) were obtained in obese adolescents with increased cardiovascular risk compared to the controls (Table 1). Significantly lower values of HDL-cholesterol were obtained in the risk group compared to the control group (p=0.001).

**Table 1:** Biochemical and anthropometric parameter values in tested subjects *Legends*: ^a^ arithmetic mean ± SD; ^b^ median and interquartile range; ^c^ Student‘s t-test; ^d^ Mann-Whitney U-test. *Abbreviations*: BMI – body mass index; WC – waist circumference; HC – hipp circumference; WHR – waist-to-hipp ratio; SOD – superoxide dismutase; GPx – glutathione peroxidase; GR – glutathione reductase; TAS –total antioxidant status; hCRP – high sensitive Creactive protein

Parameters	Group 1 (Increased risk) N=164	Group 2 (Control Group) N=74	P (significance of difference)
Age (y) ^a^	22.82±1.74	21.99±2.10	<0.001^c^
BMI (kg/m^2^) ^a^	28.78±3.89	20.60±2.04	<0.001^c^
WC (cm) M/F ^a^	97.8±10.5	73.7±6.8	<0.001^c^
HC (cm) M/F ^b^	115.0 (110–120)	98.0 (94.8–103)	<0.000 ^d^
WHpR M/F ^b^	0.85 (0.80–0.88)	0.73 (0.71–0.78)	<0.000 ^d^
SOD (U/g Hb) ^a^	1372.6±185.6	1487.1±215.3	0.0003^c^
GPx (U/g Hb) ^a^	64.8 ±10.4	68.86±13.1	0.032^c^
GR (U/L) ^a^	51.3±7.2	43.4±8.7	0.0001^c^
TAS (mmol/L) ^b^	1.42 (1.41–1.52)	1.24 (1.17–1.37)	<0.000^d^
Total cholesterol (mmol/L) ^a^	5.15±0.96	4.45±0.78	<0.0001^c^
HDL-cholesterol (mmol/L) ^a^	1.23±0.33	1.42±0.36	<0.0001^c^
LDL-cholesterol (mmol/L) ^b^	3.37 (2.72–3.89)	2.54 (2.09–3.12)	<0.0001^d^
VLDL-cholesterol (mmol/L) ^b^	0.49 (0.38–0.75)	0.34 (0.27–0.43)	<0.0001^d^
Non-HDL-cholesterol (mmol/L) ^b^	3.91 (3.1–4.6)	2.94 (2.4–3.4)	<0.0001^d^
Triglycerides (mmol/L) ^b^	1.07 (0.84–1.65)	0.74 (0.60–0.95)	<0.0001^d^
hCRP (mg/L) ^b^	2.57 (0.93–3.99)	0.56 (0.32–1.79)	0.005^d^

High sensitive C-reactive protein was significantly increased in the Group 1 compared to Group 2 (p=0.005).

A series of interesting correlations were obtained between the tested biochemical and anthropometric parameters. The anthropometric parameters correlated significantly with antioxidant markers in the whole group of examined students. BMI correlated significantly with GR (p<0.001) and TAS (p<0.001). A negative and weak correlation was obtained between SOD and HC (p=0.014), GPx and WHR (p=0.006) and between GPx and WC (p=0.02). GR and TAS correlated positively with all tested anthropometric parameters (BMI, WC, HC and WHR) (P<0.001) (Table 2).

**Table 2:** The correlations between biochemical and anthropometric parameters in the group of tested adolescents *Legends*: BMI: Body mass index; HC: hip circumference; WC: waist circumference; WHR: waist-to-hip ratio; SOD: Superoxide dismutase; GPx: Glutathione peroxidase; GR: Glutathione reductase; TAS: Total antioxidant status; hsCRP: high sensitive C-reactive protein; ρ: Spearmen’s correlation coefficient; p: significance of difference; NS-non significant

Parameters		BMI	HC	WHR	WC
SOD (U/g Hb) ρ p		-0.111 NS	-0.187 0.014	0.063 NS	-0.057 NS
GPx (U/g Hb) ρ p		-0.08 NS	-0.085 NS	-0.208 0.005	-0.163 0.028
GR (U/L) ρ p		0.456 <0.0001	0.357 0.0013	0.465 <0.0001	0.450 <0.0001
TAS (mmol/L) ρ p		0.536 <0.0001	0.469 <0.0001	0.573 <0.0001	0.584 <0.0001
hsCRP (mg/L) ρ p		0,393 0.000	0.377 0.000	0.346 0.002	0.385 0.000
Total cholesterol (mmol/L) ρ p		0.33 <0.0001	0.221 0.000	0.244 <0.0001	0.272 <0.0001
HDL-cholesterol (mmol/L) ρ p		-0.249 0.0001	-0.18 0.006	-0.274 <0.0001	-0.267 <0.0001
LDL-cholesterol (mmol/L) ρ p		0.275 <0.0001	0.174 0.007	0.198 0.002	0.212 0.001
Non-HDL-cholesterol (mmol/L) ρ p		0.392 <0.0001	0.280 <0.0001	0.301 <0.0001	0.333 <0.0001
VLDL-cholesterol (mmol/L) ρ p		0.504 <0.0001	0.437 <0.0001	0.457 <0.0001	0.503 <0.0001
Triglycerides (mmol/L) ρ p		0.51 <0.0001	0.435 <0.0001	0.467 <0.0001	0.501 <0.0001

Serum hsCRP concentration increased significantly with increased BMI (p=0.000), HC (p=0.000), WC (p=0.000) and with WHR (p=0.002) (Table 2).

The tested antioxidant parameters GR and TAS correlated positively with lipid parameters: total cholesterol, LDL-cholesterol, non-HDL-cholesterol, VLDLcho lesterol and triglycerides (P<0.05) (Table 3). Interestingly, HDL-cholesterol negatively correlated with anthropometric parameters: BMI (p<0.001), HC (p=0.007), WC (p<0.001) and WHR (p<0.001).

**Table 3:** The correlations between biochemical and lipoprotein parameters in the group of obese adolescents *Legends*: hsCRP-high senditive C-reactive protein; SOD-superoxide dismutase; GPx-glutathione peroxidase; GR-glutathione peroxidase; TAS-total antioxidant status; ρ (rho) -Spearman’s correlation coefficient; p-significance of difference; NS-non significant

Parameters	SOD	GPx	GR	TAS
Total cholesterol (mmol/L) ρ p	-0.003 NS	0.122 NS	0.262 0.02	0.239 0.04
HDL-cholesterol (mmol/L) ρ p	-0.007 NS	0.139 NS	–0.102 NS	-0.201 NS
LDL-cholesterol (mmol/L) ρ p	0.025 NS	–0.023 NS	0.246 0.03	0.296 0.015
non-HDL-cholesterol (mmol/L) ρ p	0.018 NS	0.05 NS	0.331 0.003	0.295 0.012
VLDL-cholesterol (mmol/L) ρ p	-0.026 NS	0.096 NS	0.278 0.013	0.411 0.003
Triglycerides (mmol/L) ρ p	-0.076 NS	0.093 NS	0.278 0.013	0.405 0.000
hsCRP (mg/L) ρ p	0.04 NS	0.146 NS	0.361 0.006	0.051 NS

Increased concentration of total cholesterol, LDL-, non-HDL-, and VLDL-cholesterol, and triglycerides were found with increased BMI, HC, WHTR and WC (P<0.001) (Table 2).

In the group of obese students with increased cardiovascular risk hsCRP correlated positively with SOD (r=0.389; P=0.049) and GPx (r=0.579; P=0.000). In the same group, GPx showed a weak correlation with non-HDL-cholesterol (r=0.197; P=0.04), VLDL-cholesterol (r=0.273; P=0.004) and triglycerides (r=0.268; P=0.005), while TAS correlated positively with LDL-cholesterol (r=0.455; P=0.019).

Using logistic regression analysis significant positive association was found between increased risk for cardiovascular disease and BMI (OR: 3.12; 95%CI 2.21-4.39; P=0.02), hsCRP (OR: 1.41; 95%CI 1.08-1.83; P=0.007), TAS (OR: 827.2; 95%CI 19.27-35498; P=0.007) and GR (OR: 1.13; 95%CI 1.05-1.21; P=0.002) and negative association of increased risk for CVD with GPx (OR: 0.97; 95%CI 0.94-1.003; P=0.043) and HDL-cholesterol (OR: 0.41; 95%CI 0.176-0.963; P=0.0014) (Table 4).

**Table 4:** Logistic regression analysis: the association of tested parameters with the risk for cardiovascular disease in tested group of students *Legends:* hsCRP-high sensitive C-reactive protein; BMI: Boody mass index; WC: waist circumference; HC: hip circumferemce; SOD: superoxide dismutase; GPx: glutathione peroxidase; GR: glutathione reductase; TAS: Total antioxidant status; OR: Odds ratio; CI: Confidense interval; P: significance of difference; χ^2^: chi square test

Parameter	OR	95% CI	χ^2^	P
hsCRP (mg/L)	1.41	1.08–1.83	21.02	0.007
BMI (kg/m^2^)	3.12	2.21–4.39	18.1	0.02
WC (cm)	1.44	1.29–1.60	12.33	0.137
HC (cm)	1.91	1.55–2.35	5.32	0.723
SOD (U/g Hb)	0.997	0.995–0.998	15.36	0.052
GPx (U/g Hb)	0.97	0.94–1.003	15.95	0.043
GR (U/g Hb)	1.13	1.05–1.21	23.86	0.002
TAS (mmol/L)	827.2	19.27–35498	28.70	0.0007
Total cholesterol (mmol/L)	2.31	1.64–3.26	12.37	0.135
HDL-cholesterol (mmol/L)	0.41	0.176–0.963	22.56	0.004
LDL-cholesterol (mmol/L)	2.41	1.67–3.46	9.27	0.32
Non-HDL-cholesterol (mmol/L)	2.65	1.86–3.78	12.63	0.125
VLDL-cholesterol (mmol/L)	69.1	11.9–401	14.63	0.066
Triglycerides (mmol/L)	6.95	3.14–15.39	13.2	0.105

The cutoff points of all tested parameters that could be associated with cardiovascular risk were calculated using ROC analysis (Table 5). Taking into account the statistical parameters of the ROC curve (AUC > 0.5 with 95% CI, the sensitivity and/or specificity of diagnostic tests >70%) (Table 5) and according to the determined cutoff values, we can conclude that only GR>44.8 U/L, TAS>1.35 mmol/L, hsCRP >1.71 mg/L and HDL-cholesterol<1.13 mmol/L have sufficient predictive ability for cardiovascular disease in obese students.

**Table 5:** ROC analysis for selected parameters *Legends*: AUC-area under the curve; SE-standard error; CI-confidence intervalAbrreviations: SOD – superoxide dismutase; GPx – glutathione peroxidise; GR – glutathione reductase; TAS – total antioxidant status; hCRP – high sensitive C-reactive protein

Parameter	AUC	SE	95% CI	Sensitivity %	Specificity %	Cutoff
SOD (U/g Hb)	0.660	0.071	0.53–0.77	55.6	66.7	1442
GPx (U/g Hb)	0.593	0.043	0.52–0.67	62	58.3	65
GR (U/L)	0.839	0.053	0.73–0.92	81.2	76.1	44.8
TAS (mmol/L)	0.797	0.052	0.68–0.89	92.3	67.4	1.35
hsCRP (mg/L)	0.695	0.066	0.58–0.79	73.3	75.0	1.71
Total cholesterol (mmol/L)	0.712	0.036	0.65–0.77	55.5	88.9	5.12
HDL-cholesterol (mmol/L)	0.662	0.037	0.59–0.72	48.8	81.9	1.13
LDL-cholesterol (mmol/L)	0.717	0.036	0.66–0.77	65.67	73.6	2.92
VLDL-cholesterol (mmol/L)	0.751	0.035	0.69–0.81	72.56	69.4	0.39
Non-HDL-cholesterol (mmol/L)	0.754	0.034	0.69–0.80	66.5	79.2	3.55
Triglycerides (mmol/L)	0.753	0.034	0.69–0.81	72.56	69.44	0.86

## Discussion

According to the obtained results, it can be concluded that the cardiovascular risk factors, primarily increased BMI, waist circumference, lipids (total cholesterol, LDL-cholesterol, VLDL-cholesterol, non-HDL-cholesterol, triglycerides) and inflammatory markers (hsCRP) were significantly increased in the Group 1 of University students compared to the control group (p<0.001). Numerous previous studies have shown undoubted correlation between coronary heart disease and serum lipid levels. The examination in our region, have also showed that hyperlipoproteinemia was among the most common risk factors for cardiovascular disease [17]. The analysis of cholesterol fractions and triglycerides in the students' examined groups showed that all parameters were within the reference range values, but values in the risk group were significantly increased compared to the control group (Table 1). Increased indicators of obesity (BMI and WC) in the risk group pose a risk for CVD through increased parameters of lipid status, especially WC as a cardiometabolic risk factor which has been confirmed by many studies and researches [18] [19].

It is interesting to note that some antioxidant parameters (SOD and GPX) were significantly decreased, while GR and TAS were significantly increased in the group of students with increased cardiovascular risk compared to the controls (p <0.01) although absolute values were within the reference range. CRP levels were significantly higher while HDL-cholesterol levels were significantly lower in the high-risk group compared to the control group (p = 0.006 and p<0.0001 respectively). Our results are in accordance with data reported in literature suggesting that patients with elevated waist circumference have increased oxidative stress biomarkers. Considering the association between obesity and lipid status, our finding points to the complex link between the balance of redox status and lipid profiles in young people evidenced by the study of Barbosa et al. [20]. This author points to a positive correlation between the activity of glutathione peroxidase with ox-LDLcholesterol and other lipid biomarkers. This is probably part of the antioxidant protective mechanism as some authors had shown, suggesting that increased activity of GPx can reduce the risk of cardiovascular disease while low levels of GPx can be used as independent predictor for increased risk in patients with coronary artery disease [21] [22]. Therefore, GPx may have prognostic significance along with other traditional cardiovascular risk factors. Our study showed that the most sensitive predictor for CVD in obese students was TAS. Subjects with higher TAS values than established cutoff values (1.35 mmol/L) were on average 800 times (827.2) more likely to develop cardiovascular complications compared to those students with lower TAS values. The second powerful predictor for cardiovascular events in our study was hsCRP with satisfactory sensitivity (73.3%) and specificity (75%). Obese subjects with hsCRP serum levels over 1.71 mg/L have almost 2 times higher probability to develop some cardiovascular events.

Recent publications of Nikolaidis [23] and Gutierez-Lopez [24] pointed out to altered antioxidant defense in obese people although relationship between BMI, body fat and antioxidant defense was still an open question.

The evidence of impaired obesity-related antioxidant system arises from several observational, clinical and *in vitro* studies. In a population-based study, Chrysohoou et al. [25] have documented an inverse relationship between visceral fat and total antioxidant capacity (TAC). This correlation was stronger for waist circumference with respect of BMI [25]. According to Mittal [26] and Olivares-Corichi [27], SOD, catalase and GPx activities were inversely related to BMI both in obese children and adults. Similar correlations were found in our study: SOD correlated negatively with HC (p=0.014) while GPx correlated inversely with WC and WHR (p<0.05) (Table 2). Bougoulia et al. [28] reported significant increasing activity of GPx in obese women after weight reduction. Opposite to these findings, some researchers reported that severely obese patients with greater insulin resistance have greater GPx activity than control counterparts [29]. Tabour et al. [30] demonstrated that obese patients and patients with metabolic syndrome had significantly lower TAS and higher CRP values compared to the controls (P<0.05). In that study, BMI showed a positive correlation with CRP and negative correlation with TAS. TAS correlated negatively with WC, triglycerides levels and systolic and diastolic blood pressures (P<0.001). Chrysohoou et al. [25] reported that central adiposity correlates with decreased antioxidant capacity irrespective of age, metabolic and various lifestyle variables in adults. Both Olusi et al. [31] and Ozata et al. [32] found that SOD and CPx activities were lower in obese persons as compared to non-obese individuals.

Increased cardiac lipid accumulation and altered substrate metabolism can cause cardiovascular complication in obese people. It has been documented that adipokines such as leptin, adiponectin, resistin and fatty acid binding protein 4 (FABP4) can affect cardiac structure and function in obese subjects [33].

Increased leptin concentrations are well associated with obesity and triglyceride storage. It was also documented that leptin promotes oxidative stress by increasing phagocytic activity of macrophages, inducing synthesis of pro-inflammatory cytokine (TNF-α, IL-6, IL-2) and interferon gamma (IFN-γ) and increases the levels of endothelial cell dysfunction and level of activation markers. It was hypothesized that proinflammatory effects of leptin were related to structural and functional similarities with the IL-6 family of cytokines [34].

Contributing to higher LDL-cholesterol and lower HDL-cholesterol levels, adiponectin which plays a central role in obesity-related metabolic disorders can lead to cardiac failure [35]. It has been documented that adiponectin inversely correlates with BMI and directly with TNF-α, IL-6 and CRP [36]. Increased CRP and reactive oxygen species (ROS) could trigger endothelial dysfunction that can increase the levels of ICAM-1 and VCAM-1, leading to increased monocyte chemoattraction, binding to LDL-molecules and increased risk for cardiovascular disease [37]. Metabolic disorders in obesity can disturb the oxidant-antioxidant balance which can also lead to cardiac stress. Obesity is associated with changes in antioxidant enzymes activities, altering the binding to their receptors and modifying protein functioning and their immunogenic properties. Increased oxidative stress and hyperglycemia result in accumulation of advanced glycation end products (AGE) which can cause cellular damage by inducing cross-linking of proteins and collagen and promoting alteration of extracellular matrix and vascular function and structure [38] [39].

In a clinical study conducted by Bigornia et al. [40] it has been proved that long-term weight reduction can improve cardiovascular risk by decreasing oxidative stress markers and by increasing antioxidant defense system. Diet regimen containing fruits, green vegetables, whole grains, fish and olive oil rich in monounsaturated fatty acids and Ώ-3 polyunsaturated fatty acids, vitamin C and E can reduce the weight and decrease the risk for metabolic disorders through mechanisms of cell signaling, gene-expression, decreased oxidative stress, inflammation and lipid accumulation [35].

Exercise-induced weight loss could improve the whole body redox state by modulating both oxidative stress and antioxidant biomarkers, ameliorating endothelial dysfunction and inflammation [41].

According to our results, it is obvious that obesity is strictly linked to changes in redox state. Significant association of antioxidant defense parameters with anthropometric, lipid and inflammatory markers in obese students with increased cardiovascular risk suggest that screening of these parameters is necessary and highly recommended in young adolescents especially in those with a family history of diabetes and cardiovascular disorders. It should be noted that so far, antioxidant markers have not been introduced into routine laboratory practice but are used exclusively for scientific and research purposes. The determination methods used in this study were successfully applied on Olympus AU400 analyzer which significantly shortened the determination time, reduced the reagent consumption, and increased the accuracy and precision of determination both in and between series. Therefore, methods are no longer as complicated and time-consuming as when determination is done manually. Given that most of the biochemical laboratories are today automated, each laboratory can apply these tests to the existing appliances. Introduction of these methods into daily laboratory practice would help and will allow that determination of these parameters become included into mandatory screening of obese individuals and those with increased risk for CVD. This would help such individuals to timely undergo to an adequate diet and treatment regimen which would significantly reduce morbidity and subsequent mortality from cardiovascular disease.

## Conflict of interest statement

The authors state that they have no conflicts of interest regarding the publication of this article.
